# Opioid-free anaesthesia to reduce postoperative nausea and vomiting after lower extremity wound surgery: a randomised double-blind crossover trial

**DOI:** 10.1080/07853890.2025.2517819

**Published:** 2025-06-14

**Authors:** Ya-juan Zhu, Yao-yu Ying, Hua-yue Liu, Long Qian, Hong Liu, Fu-hai Ji, Ke Peng

**Affiliations:** a Department of Anaesthesiology, First Affiliated Hospital of Soochow University, Suzhou, Jiangsu, China; b Institute of Anaesthesiology, Soochow University, Suzhou, Jiangsu, China; c Department of Medical Affairs, Second Affiliated Hospital of Soochow University, Suzhou, Jiangsu, China; d Department of Anaesthesiology, Guanyun People’s Hospital, Lianyungang, Jiangsu, China; e Department of Anaesthesiology and Pain Medicine, University of California Davis Health, Sacramento, CA, USA

**Keywords:** Lower extremity, opioid-free anaesthesia, postoperative nausea and vomiting, wound surgery

## Abstract

**Background:**

Postoperative nausea and vomiting (PONV) are common complications after surgery. Opioid use is a significant risk factor. We utilised a crossover design to test this hypothesis in the same individuals that opioid-free anaesthesia (OFA) compared with opioid-inclusive anaesthesia reduces PONV.

**Methods:**

This randomised double-blind crossover trial included adult patients undergoing two surgical procedures for lower extremity wounds under general anaesthesia. Each patient received both OFA (i.v. lidocaine, esketamine, dexmedetomidine and propofol) and opioid-inclusive anaesthesia (sufentanil and propofol); which came first was determined by randomisation. The primary outcome was the incidence of PONV during the first 48 h postoperatively. Secondary outcomes were the severity of PONV, use of rescue antiemetics, postoperative pain, need for rescue analgesia, adverse events, time to extubation, and length of recovery room stay.

**Results:**

Sixty-six patients completed this study (mean age 53 years, 36% female). The median washout period was 9 days. Compared with opioid-inclusive anaesthesia, OFA reduced the incidence of PONV 0–48 h postoperatively (5% vs. 23%, odds ratio [OR] = 0.13, 95% CI: 0.03–0.55, *p* = 0.006), which remained significant in the prespecified adjusted analysis (OR = 0.06, 95% CI: 0.01–0.32, *p* = 0.001). OFA also led to a reduced severity of PONV, a lower rate of hypotension, and a longer time to extubation. Postoperative pain and other outcomes were similar between the two anaesthetic techniques.

**Conclusion:**

This crossover trial demonstrates that OFA reduced PONV following lower extremity wound surgery, providing compelling evidence for the administration of OFA to enhance perioperative care.

**Registration:**

ChiCTR2200061511 (https://www.chictr.org.cn).

## Introduction

Patients undergoing surgery often experience postoperative nausea and vomiting (PONV), with an incidence ranging from 30% to 80% [1]. PONV episodes are distressing and associated with patient dissatisfaction, delayed recovery, a longer stay in hospital, and increased healthcare costs [1–4]. Despite many relevant studies, PONV remains an unsolved problem for surgical patients in clinical scenarios.

Opioids possess high analgesic efficacy and have long been deemed as an integral part of general anaesthesia. Nonetheless, opioid use is a major risk factor for PONV [5]. Additionally, adverse events associated with opioids (hyperalgesia, respiratory depression, delirium, immunosuppression, and physical dependence) have raised significant concerns [6–8]. Opioid-free anaesthesia (OFA) using a combination of nonopioid agents, with or without nerve blocks, has emerged to minimize opioid-related adverse effects, while providing adequate postoperative pain management [9–13].

In this study, we utilized a crossover design to test this hypothesis in the same individuals: the administration of OFA, compared with opioid-inclusive anaesthesia, would reduce the incidence of PONV during the first 48 h postoperatively in patients undergoing lower extremity wound procedures. We also evaluated the severity of PONV, postoperative pain outcomes, adverse events, and the recovery course after surgery in these patients.

## Methods

### Study design

This single-centre, randomised, double-blind, controlled crossover trial was conducted at the First Affiliated Hospital of Soochow University, Suzhou, China. Using a crossover design, each study subject served as his or her own control, that is, the same patient was tested with both OFA and opioid-inclusive anaesthesia. The original study protocol is available in Supplement 1. The trial protocol has been previously published [14].

### Ethical  approval

This study was approved by the Institutional Review Board of the First Affiliated Hospital of Soochow University (IRB #2022-108; Date of approval: April 28, 2022) and written informed consent was obtained from all subjects. The trial was registered prior to patient enrollment at Chinese Clinical Trial Registry (ChiCTR2200061511; Principal investigator: Ke Peng; Date of registration: June 27, 2022). The implementation followed the Declaration of Helsinki. The study had adhered to the Consolidated Standards of Reporting Trials (CONSORT) guidelines extension to randomised crossover trials [15].

### Patients

We included male and female patients aged ≥18 years, ASA physical status I–III, and undergoing two surgical procedures for lower extremity wounds (at least 5 days apart) under general anaesthesia. The procedures included debridement and vacuum sealing drainage for lower extremity wounds (traumatic wounds, wounds after orthopaedic surgery, diabetic foot ulcers, venous ulcers, and pressure ulcers), as described in the published study protocol [14].

Exclusion criteria were unplanned or emergency surgery, severely infected or burn wounds, sick sinus syndrome, heart rate <50 beats/min, atrioventricular block (second-degree or greater), left ventricular ejection fraction <40%, Child–Pugh grade C, renal replacement therapy, history of epilepsy or seizures, chronic use of opioids and benzodiazepines, psychiatric disease, or allergy to the study drugs.

### Randomisation and blinding

An investigational pharmacist performed online randomisation in a 1:1 ratio to randomly assign patients to one of two treatment sequences of OFA and opioid-inclusive anaesthesia (sequence 1: OFA followed by opioid-inclusive anaesthesia; sequence 2: opioid-inclusive anaesthesia followed by OFA). Using a crossover design, each patient acted as their own control with a minimum interval of 5 days between two anaesthetic treatments. Allocation details were concealed using sealed opaque envelopes and were not disclosed to patients, anaesthesia team, or research staff until trial completion. According to these sequences, the pharmacist formulated the study medications in an identical fashion. An experienced investigator who was blinded to the allocation and not involved in patient care assessed the outcome measures.

### Study interventions

For the OFA regimen, patients received i.v. lidocaine 1 mg/kg, esketamine 0.2–0.4 mg/kg and propofol 1.5–2.0 mg/kg for anaesthesia induction, followed by dexmedetomidine infusion 0.3–1.0 μg/kg/h and esketamine 0.1 mg/kg boluses as needed. For the opioid-inclusive anaesthesia, patients received intravenous sufentanil 0.2–0.4 μg/kg and normal saline with volume matched to lidocaine for anaesthesia induction, followed by normal saline infusion and sufentanil 0.1 μg/kg boluses as needed. These medication dosages were determined based on our recently published study [16].

All patients received total intravenous anaesthesia with propofol. For both anaesthetic regimens, intraoperative propofol infusion was titrated to bispectral index of 40–60. To achieve double blinding, the administration of study medications was based on our previously published protocol [14].

### Anaesthesia

The Apfel PONV risk score was calculated for each patient [5]. In the operating room, all patients received ASA standard monitoring. After induction, cisatracurium 0.2 mg/kg was given for tracheal intubation only. Upon completion of surgery, the patients were transferred to a post-anaesthesia care unit (PACU) for recovery and tracheal extubation. A spontaneous neuromuscular recovery was allowed, and pharmacological antagonism was not routinely used. In the case of residual neuromuscular blockade, patients received i.v. neostigmine 1 mg and atropine 0.5 mg. After an Aldrete score of ≥9 was reached, patients were discharged from the PACU to their surgical wards [17].

The baseline blood pressure was obtained preoperatively. During surgery and in the PACU, hypertension (increase in mean blood pressure >30% of baseline), hypotension (decrease in mean blood pressure >30% of baseline), tachycardia (heart rate >100 beats/min), and bradycardia (heart rate <50 beats/min) were treated at the discretion of the anaesthesia team. In both anaesthesia regimens, routine PONV prophylaxis included i.v. dexamethasone 5 mg and ondansetron 4 mg. Postoperative rescue antiemetic therapy with ondansetron 4 mg was administered if required. No local or regional analgesia was performed.

Postoperative multimodal analgesia comprised oral acetaminophen 500 mg every 6 h and i.v. flurbiprofen axetil 50 mg twice daily during the first two postoperative days. Rescue analgesia with i.v. nalbuphine 5 mg was administered to treat significant pain with a numerical rating scale (NRS, ranging 0–10; 0 represents no pain and 10 represents the most severe pain) score ≥4. Other perioperative care was provided according to the institutional clinical standards.

### Study outcomes and data collection

The primary outcome was the incidence of PONV during the first 48 h after surgery. Secondary outcomes were the severity of PONV (in the PACU, from PACU discharge to 24 h postoperatively, and within 24–48 h after surgery), need for rescue antiemetics, postoperative pain, need for rescue analgesics, adverse events, time to extubation, and length of PACU stay.

The severity of PONV was assessed using the simplified PONV impact scale (ranging 0–6; a higher score indicates a greater severity) [9,16,18,19]. If a patient had a score of 1 or above, they were counted as having PONV. Postoperative NRS pain scores were assessed at PACU discharge and 24 and 48 h postoperatively, and patients were also asked to rate their worst pain. An investigator blinded to the treatment sequences assessed the outcome measures *via* ward visits or telephone after hospital discharge. Hemodynamic events, use of rescue antiemetics and analgesics, postoperative sedation (Richmond Agitation Sedation Scale score ≤ −2), hypoxemia (peripheral oxygen saturation <90% in room air), psychotomimetic or dissociative effects, time to extubation, length of PACU stay, and other perioperative data were collected in the DoCare Anaesthesia Clinical Information System (V5.0; Suzhou MedicalSystem Co., Ltd, Suzhou, China) and Electronic Medical Records (Nanjing HaiTai Medical Information System Co., Ltd, Nanjing, China).

### Sample size calculation

According to previous studies [11,12], the rate of PONV was approximately 30% in surgical patients receiving opioid-inclusive anaesthesia. Our recent studies showed that OFA reduced the incidence of PONV from 32% to 15% (53% reduction in relative risk) after thoracoscopic lung surgery [19], and from 24% to 5% (79% reduction in relative risk) after thyroid and parathyroid surgery [16]. Based on these, we assumed that a 20% absolute reduction in the incidence of PONV (66% reduction in relative risk) would be reasonable. To provide an 80% power at a two-sided α level of 0.05, a sample size of 59 participants was needed (PASS; version 15.0.5, NCSS, LCC, Kaysville, UT, USA). Considering a possible drop-out rate of 18% (due to consent withdrawn, surgery cancelled or the second surgical procedure not performed, lost to follow-up, or unavailable research staff), the sample size was expanded to 72 patients.

### Statistical analysis

The normality of continuous variables was assessed using the Shapiro–Wilk test. Normally distributed variables are presented as means (standard deviations [SDs]), whereas non-normally distributed variables are shown as medians (interquartile ranges [IQRs]). Categorical variables are expressed as *n* (%).

Demographic and baseline data were analyzed using descriptive statistics only. The dose of propofol and length of surgery were analyzed using the Mann–Whitney test. The primary and secondary outcomes were analyzed using a logistic regression, a mixed linear model, or an ordinal model (with treatment, sequence, and period as fixed effects and subject as a random effect). The effect size was assessed using the odds ratio (OR) or difference with 95% confidence interval (CI). In addition, the treatment effect was adjusted for Apfel PONV risk scores and length of surgery. In addition, a prespecified subgroup analysis for the primary outcome was conducted according to sex (female vs. male) and Apfel PONV risk scores (0–1 vs. 2–3).

All analyses were conducted on a modified intention-to-treat basis (including all randomised patients who completed two surgical procedures with available outcome data). We did not perform imputation for missing values. The secondary outcomes were not adjusted for multiple testing and should be interpreted as exploratory. Statistical procedures were performed using the R software (version 3.6.0, R Foundation for Statistical Computing, Vienna, Austria). Two-sided *p* values <0.05 were considered statistically significant.

## Results

### Patient and perioperative characteristics

**Figure 1.**
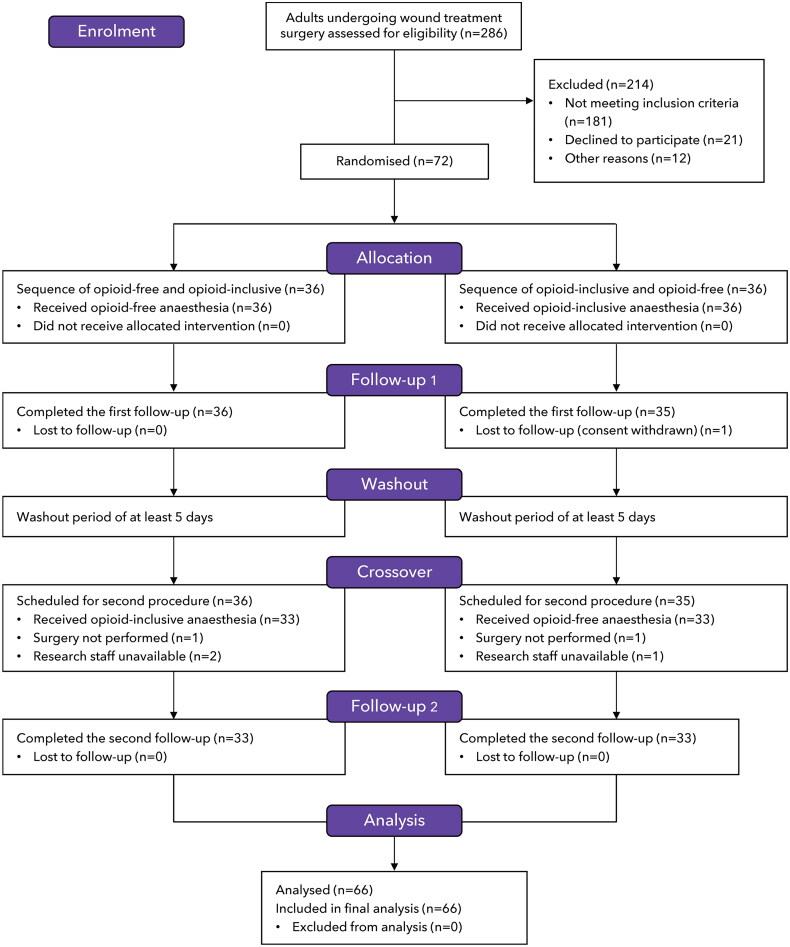
CONSORT study flow diagram.

Between June and December 2022, a total of 286 patients were screened (Figure 1). Of these, 72 patients were randomly assigned to one of two treatment sequences. Six patients dropped out of this study due to withdrawal of consent, cancelled surgery, or unavailable research staff. Finally, 66 patients completed both the crossover sessions.

Table 1 shows the demographic and baseline data. For all included patients, the mean (SD) age was 52.5 (14.1) years, and 36% of patients were female. Most patients were at ASA physical status I and II. For all patients, two standardised surgical procedures (debridement followed by vacuum sealing drainage placement) were performed for lower extremity wounds of the same etiology by the same surgical team. The median (IQR) surgical time was 75 (55–100) min for the first procedure and 80 (55–105) min for the second procedure (*p* = 0.579). The median (IQR) washout phase was 9 (7–14) days.

**Table 1.** Demographic and baseline data.

	All patients (*n* = 66)	Opioid-free first (*n* = 33)	Opioid-inclusive first (*n* = 33)
Age (years)	52.5 (14.1)	57 (11.6)	48 (15.1)
Female	24 (36%)	15 (45%)	9 (27%)
Weight (kg)	66 (11.7)	64.4 (11.7)	67.5 (11.7)
Height (cm)	166.2 (7.6)	164.8 (7.7)	167.7 (7.3)
BMI (kg/m^–2^)	23.8 (3.5)	23.7 (3.9)	23.9 (3.2)
Nonsmoker	42 (64%)	24 (73%)	18 (55%)
History of PONV or motion sickness	12 (18%)	7 (21%)	5 (15%)
ASA physical status			
I	14 (21%)	5 (15%)	9 (27%)
II	45 (68%)	25 (76%)	20 (61%)
III	7 (11%)	3 (9%)	4 (12%)
Apfel PONV risk score			
0	24 (36%)	9 (27%)	15 (45%)
1	17 (26%)	9 (27%)	8 (24%)
2	15 (23%)	9 (27%)	6 (18%)
3	10 (15%)	6 (18%)	4 (12%)
Total risk scores	1.2 (1.1)	1.4 (1.1)	1 (1.1)
Comorbidities			
Hypertension	17 (26%)	9 (27%)	8 (24%)
Diabetes	17 (26%)	6 (18%)	11 (33%)
History of stroke	5 (8%)	3 (9%)	2 (6%)
Arrhythmia	3 (5%)	3 (9%)	0
Other	2 (3%)	2 (6%)	0
Preoperative medications			
ACEI/ARB	5 (8%)	3 (9%)	2 (6%)
Calcium channel blocker	11 (17%)	4 (12%)	7 (21%)
Beta blocker	1 (2%)	0	1 (3%)
Insulin	9 (14%)	3 (9%)	6 (18%)
Antidiabetic drug	8 (12%)	3 (9%)	5 (15%)
Aspirin	3 (5%)	2 (6%)	1 (3%)
Surgical type			
Traumatic wounds	30 (45%)	15 (45%)	15 (45%)
Wounds after orthopaedic surgery	18 (27%)	10 (30%)	8 (24%)
Diabetic foot	5 (8%)	2 (6%)	3 (9%)
Other	13 (20%)	6 (18%)	7 (21%)
Washout period (days)	9 (7–14)	12 (7–14)	8 (7–14)
5–10 days	37 (56%)	16 (48%)	21 (64%)
11–15 days	17 (26%)	9 (27%)	8 (24%)
> 15 days	12 (18%)	8 (24%)	4 (12%)

Values are mean (SD), median (IQR), or *n* (%).

PONV, postoperative nausea and vomiting; ACEI/ARB, angiotensin converting enzyme inhibitor/angiotensin receptor antagonist.

Patients received a median (IQR) dose of esketamine 50 (40–50) mg, lidocaine 65 (60–74) mg, and dexmedetomidine 42 (35–55) μg during OFA, and sufentanil 50 (40–60) μg during opioid-inclusive anaesthesia. The median (IQR) propofol doses were 735 (600–900) mg and 755 (628–873) mg during OFA and opioid-inclusive anaesthesia, respectively (*p* = 0.820). The median (IQR) length of surgery was 80 (60–105) min with OFA and 75 (55–95) min with opioid-inclusive anaesthesia (*p* = 0.448).

### Primary outcome

**Figure 2.**
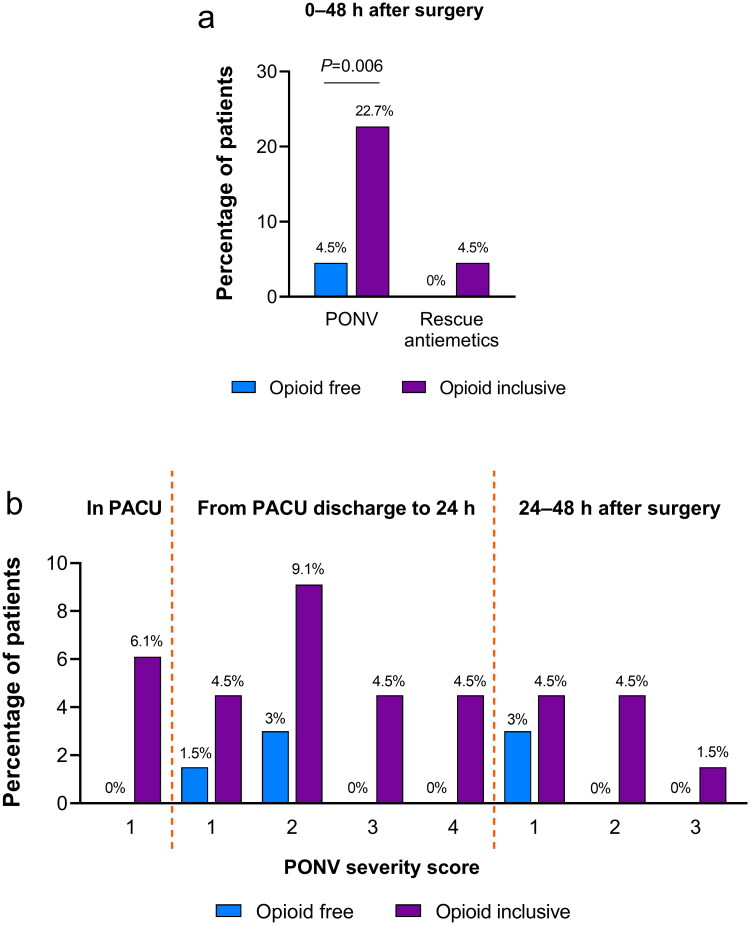
Postoperative nausea and vomiting outcomes. (a) Percentage of patients with PONV and rescue antiemetics during 0–48 h after surgery. (b) Percentage of patients with different PONV severity scores in PACU, from PACU discharge to 24 h, and during 24–48 h after surgery. PACU, post-anaesthesia care unit; PONV, postoperative nausea and vomiting.

OFA significantly reduced the incidence of PONV during the first 48 h after surgery compared with opioid-inclusive anaesthesia (5% vs. 23%; OR = 0.13, 95% CI: 0.03–0.55; *p* = 0.006) (Table 2; Figure 2a). After prespecified adjustment for Apfel risk scores and length of surgery, OFA still led to a lower rate of PONV (OR = 0.06, 95% CI: 0.01–0.32; *p* = 0.001). The effect of OFA on PONV did not differ in the prespecified subgroups of sex or Apfel PONV risk scores (Table 3).

**Table 2.** Primary and secondary outcomes.

	Opioid-free (*n* = 66)	Opioid-inclusive (*n* = 66)	Effect size (95% CI)^a^	*p* value^a^	Adjusted effect size (95% CI)^b^	Adjusted *P* value^b^
**Primary outcome**						
PONV 0–48 h	3 (5%)	15 (23%)	0.13 (0.03, 0.55)	0.006	0.06 (0.01, 0.32)	0.001
**Secondary outcomes**						
PONV severity in PACU			–	–	–	–
0	66 (100%)	62 (94%)				
1	0	4 (6%)				
PONV severity PACU–24 h			−0.2 (–0.5, 0)	0.006	−0.1 (–0.2, 0)	< 0.001
0	63 (96%)	51 (77%)				
1	1 (2%)	3 (5%)				
2	2 (3%)	6 (9%)				
3	0	3 (5%)				
4	0	3 (5%)				
PONV severity 24–48 h			−0.3 (–1.1, 0.1)	0.099	−0.2 (–0.9, 0)	0.049
0	64 (97%)	59 (89%)				
1	2 (3%)	3 (5%)				
2	0	3 (5%)				
3	0	1 (2%)				
Rescue antiemetics 0–48 h	0	3 (5%)	–	–	–	–
Pain at PACU discharge	1.9 (0.9)	1.9 (0.8)	0 (–0.2, 0.3)	0.738	0 (–0.2, 0.3)	0.739
Pain at 24 h	3.0 (1.5)	3.1 (1.1)	−0.1 (–0.5, 0.3)	0.599	−0.1 (–0.5, 0.3)	0.598
Pain at 48 h	2.2 (1.0)	2.2 (1.1)	0 (–0.2, 0.4)	0.698	0 (–0.2, 0.4)	0.697
The worst pain	3.3 (1.4)	3.4 (1.1)	−0.1 (–0.5, 0.2)	0.514	−0.1 (–0.5, 0.3)	0.598
Rescue analgesia 0–48 h	6 (9%)	9 (14%)	0.4 (0.1, 2.1)	0.305	0.4 (0.1, 2.1)	0.270
Hypertension	4 (6%)	2 (3%)	–	–	–	–
Tachycardia	2 (3%)	1 (2%)	–	–	–	–
Hypotension	12 (18%)	23 (35%)	0.38 (0.16, 0.93)	0.033	0.38 (0.16, 0.93)	0.034
Bradycardia	3 (5%)	0	–	–	–	–
Postoperative sedation	2 (3%)	3 (5%)	–	–	–	–
Hypoxemia	0	0	–	–	–	–
Psychotomimetic or dissociative effects	5 (8%)	1 (2%)	–	–	–	–
Time to extubation; min	12 (5.7)	9.4 (5.8)	2.6 (0.8, 4.4)	0.005	2.6 (0.8, 4.4)	0.005
Length of PACU stay; min	36.3 (14.8)	34.5 (13.7)	1.8 (–2.9, 6.6)	0.445	1.8 (–2.8, 6.5)	0.439

Values are mean (SD) or *n* (%).

PONV, postoperative nausea and vomiting; PACU, post-anesthesia care unit.

^a^Effect size (odds ratio or difference) analyzed using a logistic regression, a mixed linear model, or an ordinal model with treatment, sequence, and period as fixed effects and subject as a random effect.

^b^Adjusted for Apfel PONV risk scores and length of surgery.

**Table 3.** Subgroup analysis of the primary outcome.

	Opioid-free (*n* = 66)	Opioid-inclusive (*n* = 66)	Odds ratio (95% CI)	*p* value for interaction
Sex				
Female	3/24 (13%)	13/24 (54%)	0.05 (0.00, 0.75)	0.973
Male	0/42	2/42 (5%)	0.00 (0.00, 5.30)	
Apfel PONV risk score				
0–1	0/41	2/41 (5%)	0.00 (0.00, 5.30)	0.943
2–3	3/25 (12%)	13/25 (52%)	0.05 (0.00, 0.66)	

Values are *n* (%).

PONV, postoperative nausea and vomiting.

### Secondary outcomes

Details of PONV episodes are presented in Table 2 and Figure 2b. OFA significantly reduced PONV severity until 24 h after surgery and during 24–48 h after surgery. No rescue antiemetics were needed after OFA, whereas three patients required antiemetic rescue therapy after opioid-inclusive anaesthesia. Postoperative pain, need for rescue analgesia, sedation scores, and length of PACU stay were similar between the two anaesthetic regimens. OFA led to a lower incidence of hypotension. Hypertension, tachycardia, bradycardia, and psychotomimetic or dissociative effects were uncommon. The time to extubation was slightly longer after OFA.

## Discussion

In this randomised, double-blind, controlled, crossover trial, OFA compared with opioid-inclusive anaesthesia significantly reduced the incidence of PONV in patients undergoing repeated lower extremity wound surgeries. Based on a crossover design, the same individuals underwent two surgical procedures and received both anaesthetic regimens: one procedure was performed with OFA and the other with opioid-inclusive anaesthesia. The beneficial effect of OFA on decreasing PONV was still evident in the prespecified analysis after adjusting for Apfel PONV risk scores and length of surgery. OFA also mitigated the severity of PONV and reduced the incidence of hypotension, while slightly prolonging the time to extubation. Postoperative pain control was adequate in both groups.

Many opioid-sparing and opioid-free anaesthetic regimens are currently being investigated, with the primary aim of reducing opioid-related adverse effects. The concept of OFA refers to avoiding opioid use during anaesthesia while maintaining adequate postoperative pain control [20]. OFA is a ‘radical’ form of an opioid-sparing anaesthesia, and the feasibility of OFA has been demonstrated in various surgical procedures [9–13,16,19,21–23]. Recently, we have conducted two randomised controlled trials: one study found that OFA with esketamine, dexmedetomidine and sevoflurane reduced PONV after thoracoscopic lung surgery (15% vs. 32%) [19]; another study showed that OFA with esketamine, lidocaine, dexmedetomidine and propofol led to a lower rate of PONV (5% vs. 24%) after thyroid and parathyroid surgery [16]. Here, this randomised crossover trial further confirmed the reduction of PONV by the OFA regimen for surgical patients. Moreover, our stratified analyses suggested that OFA may offer more significant prophylactic effect for patients at increased risks of PONV, such as female patients (13% vs. 54%) and patients with higher Apfel PONV risk scores (12% vs. 52%). Thus, our findings underscore the clinical implications of using the OFA regimen for patients with high-risk profiles for PONV.

It is well known that the use of opioids induces nausea and vomiting [24]. OFA not only reduces the incidence of PONV by omitting intraoperative opioids, but also the drugs that are used in the OFA per se have antiemetic effects. Intravenous lidocaine may exert anti-PONV effects by accelerating gastrointestinal recovery and its anti-inflammatory action [25,26]. Dexmedetomidine could inhibit sympathetic outflow, modulate the release of neurotransmitters (5-hydroxytryptamine and dopamine), and suppress histamine-induced interleukin-6, thereby reducing PONV [27,28]. In this study, esketamine was administered at low doses (0.2–0.4 mg/kg) in combination with lidocaine and dexmedetomidine, which effectively mitigated its potential side effects (nausea, vomiting, increased secretions, and psychotomimetic effect) while preserving its analgesic benefits. Additionally, esketamine may reduce the levels of pro-inflammatory cytokines (peripheral emetic signals), thereby mitigating postoperative intestinal paralysis and nausea-related intestinal dysfunction [29,30].

Regarding the safety of OFA, we found that the incidence of hypotension was reduced when patients received OFA, without an increased risk of bradycardia (the most significant adverse event associated with OFA). There are two possible reasons for these findings. First, our patients received intraoperative dexmedetomidine infusion at a rate of 0.3–1.0 μg/kg/h without the use of a loading dose, thereby minimising its common haemodynamic adverse effects (i.e. hypotension and bradycardia). Second, the combined use of esketamine contributed to better haemodynamic stability during anaesthesia. Esketamine activates the sympathetic nervous system to maintain blood pressure and counter respiratory depression [24,31,32]. Our results are consistent with a recent study, in which OFA administration reduced the rate of hypotension with intervention during thyroid and parathyroid surgery [16]. In addition, we did not observe significant differences in other adverse events including hypertension, tachycardia, postoperative sedation, and psychotomimetic or dissociative effects between the two anaesthetic regimens. The extubation time after OFA was slightly longer than that after opioid-inclusive anaesthesia, with a median difference of 2.6 min. This result was statistically significant but not clinically meaningful, as the length of PACU stay was similar between the two anaesthesia regimens.

Owing to a crossover design, the difference between the two anaesthetic regimens was derived from a within-subject comparison, which removed the inter-subject variability and diminished the confounding effects of covariates. Another advantage of the crossover design is the increased statistical power and efficiency compared with the parallel design [33]. On the other hand, a major consideration regarding the crossover design is the carryover effect from the first treatment. If a crossover effect is present, it is difficult to interpret the study results. In our study, the median washout period was nine days. During this period, we believed that the effects of the first anaesthetic regimen and other medications subsided completely when patients underwent a second surgical procedure and anaesthesia.

We acknowledge some limitations of this trial. First, we used a specific OFA protocol that has been applied in our recent study [16]. Nonetheless, the optimal method to deliver OFA for patients undergoing various surgical procedures warrants further research. Second, it is possible that there were occasions where the same anaesthesiologists performed both interventions for the same patients. This might introduce some performance bias, but PONV is an objective outcome measure and postoperative assessors were blinded to the treatment sequence assignment. Third, it is also possible that the two surgeries performed in the same patient were not exactly the same, and thus patients’ responses may not be the same. However, we showed that patients in the two anaesthesia regimens received the same amount of propofol, and the lengths of surgery were also similar between the two types of anaesthesia. In addition, we designed this crossover approach by studying the same patients under a decent washout period and utilized the statistical methods in adjusting analysis for Apfel PONV risk scores and duration of surgery. Last, this single-centre study was carried out in eastern China. For patients in Western countries, the extent to which the OFA regimen alters the incidence and severity of PONV should be explored in future research.

## Conclusions

This randomised crossover trial demonstrates that the OFA regimen with i.v. lidocaine, esketamine, dexmedetomidine and propofol, compared with opioid-inclusive anaesthesia, yielded a significantly lower incidence of PONV in the same individuals after lower extremity wound surgery. Our results provide compelling evidence for the implementation of OFA to mitigate PONV and enhance perioperative care in patients undergoing surgery.

## Supplementary Material

CONSORT Checklist.pdf

Supplement.docx

## Data Availability

The data that support the findings of this study are available from the corresponding author upon reasonable request.

## References

[1] Gan TJ, Belani KG, Bergese S, et al. Fourth consensus guidelines for the management of postoperative nausea and vomiting. Anesth Analg. 2020;131(2):411–448. doi: 10.1213/ANE.0000000000004833. 32467512

[2] Markwei MT, Babatunde IO, Kutlu-Yalcin E, et al. Perioperative supplemental oxygen and postoperative nausea and vomiting: subanalysis of a trial, systematic review, and meta-analysis. Anesthesiology. 2023;138(1):56–70. doi: 10.1097/ALN.0000000000004428. 36480644

[3] Lavand’homme P, Kehlet H. Benefits versus harm of intraoperative glucocorticoid for postoperative nausea and vomiting prophylaxis. Br J Anaesth. 2023;131(1):8–10. doi: 10.1016/j.bja.2023.04.013. 37183100

[4] Benhamou D. Postoperative nausea and vomiting: is the big little problem becoming a smaller little problem? Br J Anaesth. 2023;131(1):22–25. doi: 10.1016/j.bja.2023.04.004. 37179157

[5] Apfel CC, Läärä E, Koivuranta M, et al. A simplified risk score for predicting postoperative nausea and vomiting: conclusions from cross-validations between two centers. Anesthesiology. 1999;91(3):693–700. doi: 10.1097/00000542-199909000-00022. 10485781

[6] Sun Q, Li Z, Wang Z, et al. Immunosuppression by opioids: mechanisms of action on innate and adaptive immunity. Biochem Pharmacol. 2023;209:115417. doi: 10.1016/j.bcp.2023.115417. 36682388

[7] Yiu CH, Gnjidic D, Patanwala A, et al. Opioid-related adverse drug events in surgical patients: risk factors and association with clinical outcomes. Expert Opin Drug Saf. 2022;21(9):1211–1223. doi: 10.1080/14740338.2022.2049230. 35234566

[8] Shanthanna H, Ladha KS, Kehlet H, et al. Perioperative opioid administration. Anesthesiology. 2021;134(4):645–659. doi: 10.1097/ALN.0000000000003572. 32991672

[9] Massoth C, Schwellenbach J, Saadat-Gilani K, et al. Impact of opioid-free anaesthesia on postoperative nausea, vomiting and pain after gynaecological laparoscopy – a randomised controlled trial. J Clin Anesth. 2021;75:110437. doi: 10.1016/j.jclinane.2021.110437. 34229292

[10] Beloeil H, Garot M, Lebuffe G, et al. Balanced opioid-free anesthesia with dexmedetomidine versus balanced anesthesia with remifentanil for major or intermediate noncardiac surgery. Anesthesiology. 2021;134(4):541–551. doi: 10.1097/aln.0000000000003725. 33630043

[11] Bakan M, Umutoglu T, Topuz U, et al. Opioid-free total intravenous anesthesia with propofol, dexmedetomidine and lidocaine infusions for laparoscopic cholecystectomy: a prospective, randomized, double-blinded study. Braz J Anesthesiol. 2015;65(3):191–199. doi: 10.1016/j.bjane.2014.05.001. 25925031

[12] Ziemann-Gimmel P, Goldfarb AA, Koppman J, et al. Opioid-free total intravenous anaesthesia reduces postoperative nausea and vomiting in bariatric surgery beyond triple prophylaxis. Br J Anaesth. 2014;112(5):906–911. doi: 10.1093/bja/aet551. 24554545

[13] Léger M, Perrault T, Pessiot-Royer S, et al. Opioid-free anesthesia protocol on the early quality of recovery after major surgery (SOFA trial): a randomized clinical trial. Anesthesiology. 2024;140(4):679–689. doi: 10.1097/ALN.0000000000004840. 37976460

[14] Zhu YJ, Wang D, Long YQ, et al. Effects of opioid-free total intravenous anesthesia on postoperative nausea and vomiting after treatments of lower extremity wounds: protocol for a randomized double-blind crossover trial. Perioper Med (Lond). 2023;12(1):38. doi: 10.1186/s13741-023-00329-9. 37452385 PMC10347776

[15] Dwan K, Li T, Altman DG, et al. CONSORT 2010 statement: extension to randomised crossover trials. BMJ. 2019;366:l4378. doi: 10.1136/bmj.l4378. 31366597 PMC6667942

[16] Wang D, Sun Y, Zhu YJ, et al. Comparison of opioid-free and opioid-inclusive propofol anaesthesia for thyroid and parathyroid surgery: a randomised controlled trial. Anaesthesia. 2024;79(10):1072–1080. doi: 10.1111/anae.16382. 39037325

[17] Aldrete JA. Post-anesthetic recovery score. J Am Coll Surg. 2007;205(5):e3-4–e4-5. doi: 10.1016/j.jamcollsurg.2007.07.034. 17964430

[18] Myles PS, Wengritzky R. Simplified postoperative nausea and vomiting impact scale for audit and post-discharge review. Br J Anaesth. 2012;108(3):423–429. doi: 10.1093/bja/aer505. 22290456

[19] Feng C, Xu Y, Chen S, et al. Opioid-free anaesthesia reduces postoperative nausea and vomiting after thoracoscopic lung resection: a randomised controlled trial. Br J Anaesth. 2024;132(2):267–276. doi: 10.1016/j.bja.2023.11.008. 38042725

[20] Chia PA, Cannesson M, Bui CCM. Opioid free anesthesia: feasible? Curr Opin Anaesthesiol. 2020;33(4):512–517. doi: 10.1097/ACO.0000000000000878. 32530891 PMC7502015

[21] Chassery C, Atthar V, Marty P, et al. Opioid-free versus opioid-sparing anaesthesia in ambulatory total hip arthroplasty: a randomised controlled trial. Br J Anaesth. 2024;132(2):352–358. doi: 10.1016/j.bja.2023.10.031. 38044236

[22] Zhou F, Cui Y, Cao L, Opioid-Free Anesthesia Working G. The effect of opioid-free anaesthesia on the quality of recovery after endoscopic sinus surgery: a multicentre randomised controlled trial. Eur J Anaesthesiol. 2023;40(8):542–551. doi: 10.1097/EJA.0000000000001784. 37377372

[23] Hao C, Xu H, Du J, et al. Impact of opioid-free anesthesia on postoperative quality of recovery in patients after laparoscopic cholecystectomy: a randomized controlled trial. Drug Des Devel Ther. 2023;17:3539–3547. doi: 10.2147/DDDT.S439674. PMC10693280 38046284

[24] Jonkman K, van Rijnsoever E, Olofsen E, et al. Esketamine counters opioid-induced respiratory depression. Br J Anaesth. 2018;120(5):1117–1127. doi: 10.1016/j.bja.2018.02.021. 29661389

[25] Nakajima D, Kawakami H, Mihara T, et al. Effectiveness of intravenous lidocaine in preventing postoperative nausea and vomiting in pediatric patients: a systematic review and meta-analysis. PLoS One. 2020;15(1):e0227904. doi: 10.1371/journal.pone.0227904. 31990953 PMC6986726

[26] Dunn LK, Durieux ME. Perioperative use of intravenous lidocaine. Anesthesiology. 2017;126(4):729–737. doi: 10.1097/ALN.0000000000001527. 28114177

[27] Zhao W, Li J, Wang N, et al. Effect of dexmedetomidine on postoperative nausea and vomiting in patients under general anaesthesia: an updated meta-analysis of randomised controlled trials. BMJ Open. 2023;13(8):e067102. doi: 10.1136/bmjopen-2022-067102. PMC10394554 37527891

[28] Chen Y, Li M, Zheng Y, et al. The preventive effect of dexmedetomidine on anesthesia complications in strabismus surgery: a systematic review and meta-analysis. BMC Anesthesiol. 2023;23(1):253. doi: 10.1186/s12871-023-02215-9. 37491215 PMC10367359

[29] Zhao L, Li Z, Jin B, et al. Safety and efficacy of low-dose esketamine in laparoscopic cholecystectomy: a prospective, double-blind randomized controlled trial. BMC Anesthesiol. 2024;24(1):47. doi: 10.1186/s12871-024-02429-5. 38302944 PMC10832235

[30] Han L, Tian B, Li S. Esketamine has promising anti-inflammatory effects in orthopedic surgery and plays a protective role in postoperative cognitive function and pain management. Am J Transl Res. 2025;17(1):277–285. doi: 10.62347/VTKD5295. 39959204 PMC11826177

[31] Song N, Yang Y, Zheng Z, et al. Effect of esketamine added to propofol sedation on desaturation and hypotension in bidirectional endoscopy: a randomized clinical trial. JAMA Netw Open. 2023;6(12):e2347886. doi: 10.1001/jamanetworkopen.2023.47886. 38117498 PMC10733809

[32] Li N, Qi X, Bao J, et al. A comparative study of esketamine-propofol and sufentanil-propofol for analgesia and sedation during breast minimally invasive rotary resection with local anesthesia: a randomized double-blind clinical trial. Drug Des Devel Ther. 2024;18:5397–5407. doi: 10.2147/DDDT.S487872. PMC11606144 39618428

[33] Lim CY, In J. Considerations for crossover design in clinical study. Kor J Anesthesiol. 2021;74(4):293–299. doi: 10.4097/kja.21165. PMC8342834 34344139

