# Supplementary material for: Opioid-free anaesthesia to reduce postoperative nausea and vomiting after lower extremity wound surgery: a randomised double-blind crossover trial
Source: Ann Med. 2025 Jun 14;57(1):2517819. doi: 10.1080/07853890.2025.2517819 (PMC12168384; doi:10.1080/07853890.2025.2517819)
Supplement: Supplement.docx [file IANN_A_2517819_SM1374.docx]

**Effects of opioid-free propofol anesthesia on postoperative nausea and vomiting after treatments of lower extremity wounds**

*A randomized double-blind controlled crossover trial*

| **Title** | Effects of opioid-free propofol anesthesia on postoperative nausea and vomiting after treatments of lower extremity wounds |
| --- | --- |
| **Institution** | The First Affiliated Hospital of Soochow University |
| **Principal Investigator** | Ke Peng, MD, PhD |
| **Address of Principle Investigator** | Department of Anesthesiology  The First Affiliated Hospital of Soochow University  188 Shizi St, Suzhou, Jiangsu, 215006, China  Tel: +86-13656207331  Email: pengke0422@163.com; pengke@suda.edu.cn |
| **Concept and Design** | Ke Peng, Ya-juan Zhu, Fu-hai Ji |
|  |  |
| **Study Contact** | Ke Peng  Address: 188 Shizi Street, Suzhou, Jiangsu, 215006, China  Tel: +86-15962155989  Email: pengke0422@163.com; pengke@suda.edu.cn |
| **Version**  **Date** | 1.0  2022-04-22 |

**Table of Contents**

[1. Synopsis 4](#_Toc130496217)

[2. Flow Chart 6](#_Toc130496218)

[3. Glossary 7](#_Toc130496219)

[4. Background and Rationale 9](#_Toc130496220)

[4.1 Postoperative nausea and vomiting 9](#_Toc130496221)

[4.2 Opioid-free anesthesia 9](#_Toc130496222)

[4.3 The aim of this study 9](#_Toc130496223)

[5. Study Design 10](#_Toc130496224)

[5.1 Description of this study 10](#_Toc130496225)

[5.2 Duration of this study 10](#_Toc130496226)

[6. Study Outcomes 11](#_Toc130496227)

[6.1 Primary outcome 11](#_Toc130496228)

[6.2 Secondary outcomes 11](#_Toc130496229)

[7. Selection of Patients 12](#_Toc130496231)

[7.1 Inclusion criteria 12](#_Toc130496232)

[7.2 Exclusion criteria 12](#_Toc130496233)

[8. Study Treatment 13](#_Toc130496234)

[8.1 Randomization and blinding 13](#_Toc130496235)

[8.2 Anesthesia and study interventions 13](#_Toc130496236)

[8.3 Accountability and compliance 14](#_Toc130496237)

[9. Safety Concerns 15](#_Toc130496239)

[9.1 Safety profile of opioid-free anesthesia 15](#_Toc130496240)

[9.2 Perioperative monitoring 15](#_Toc130496241)

[9.3 Discontinuation of intervention and unmasking 15](#_Toc130496242)

[10. Statistical Plan 17](#_Toc130496243)

[10.1 Sample size 17](#_Toc130496244)

[10.2 Statistical analysis 17](#_Toc130496245)

[10.3 Statisticians 18](#_Toc130496246)

[11. Ethical Considerations 19](#_Toc130496247)

[11.1 Ethical approval and clinical trial registration 19](#_Toc130496248)

[11.2 Informed consent 19](#_Toc130496249)

[11.3 Responsibilities 19](#_Toc130496250)

[12. Data Management and Clinical Trial Report 20](#_Toc130496251)

[12.1 Source documents 20](#_Toc130496252)

[12.2 Case Report Forms (CRFs) 20](#_Toc130496253)

[12.3 Data protection 20](#_Toc130496254)

[12.4 Clinical trial report 20](#_Toc130496255)

[13. References 21](#_Toc130496256)

# **Synopsis**

| **Study Title** | Effects of opioid-free propofol anesthesia on postoperative nausea and vomiting after treatments of lower extremity wounds |
| --- | --- |
| **Study Design** | Researcher-initiated, single-center, randomized, double-blind, placebo-controlled, crossover trial |
| **Principal Investigator** | Ke Peng, MD, PhD |
| **Trial Site** | The First Affiliated Hospital of Soochow University |
| **Selection Criteria** | **Inclusion Criteria:**   1. Age ≥ 18 years old; 2. American society of Anesthesiologists status I to III; 3. Scheduled for two separate treatments for lower extremity wounds under general anesthesia; 4. An interval between the procedures ≥ 5 days.   **Exclusion Criteria:**   1. Unplanned or emergency surgery; 2. Severely infected or burn wounds; 3. Left ventricular ejection fraction < 40%, second-degree or greater atrioventricular block, sick sinus syndrome, or severe bradycardia (heart rate < 50 beats/min); 4. Severe liver or renal dysfunction (Child Pugh grade C or renal replacement therapy); 5. Epilepsy or seizures; 6. Preoperative use of sedatives or analgesics; 7. Allergy to medications used in this study. |
| **Study Outcomes** | **Primary outcome:**  The incidence of postoperative nausea and vomiting (PONV) within the first 48 h postoperatively |
|  | **Secondary outcomes:**   - Severity of PONV within 48 h postoperatively; - Antiemetic rescue therapy; - Postoperative pain scores within 48 h postoperatively, the worst pain, and need for rescue analgesia; - Postoperative sedation; - Hypotension, bradycardia, hypertension, tachycardia, and hypoxemia; - Psychotomimetic or dissociative effects; - Time to extubation; - Length of post-anesthesia care unit stay. |
| **Expected Number of Patients** | 72 adult patients |
| **Study Drug and Interventions** | Patients will be randomized (1:1) to 1 of 2 treatment sequences: opioid-free followed by opioid-inclusive anesthesia (sequence 1) and opioid-inclusive followed by opioid-free anesthesia (sequence 2). |
| **Safety Concerns** | The administration and dosage of study medications in both anesthesia regimens are within the current clinical practice, we believe that serious adverse events should be rare.   - Esketamine may induce psychiatric adverse effects (dizziness, headache, hallucination, nightmare, dissociation, emergence delirium, or illusions), but most studies found these effects to be self-limiting and clinically benign. - To avoid potential adverse effects, a high dose of esketamine or dexmedetomidine will be avoided. |
| **Statistical Plan** | The treatment effects will be assessed using the odds ratio (OR) or difference in means/medians with 95% confidence intervals (CI). A mixed linear model or logistic regression model will be used to test for treatment differences with sequence, period, and treatment as fixed effects and subject as a random effect.  Additional analyses will be performed adjusting for two covariates (Apfel’s PONV risk scores and length of procedures). Prespecified subgroup analyses will be conducted for the primary outcome according to sex and PONV risk scores. Neither an interim analysis nor an imputation of missing data is planned. We have no plan of multiple testing correction for the secondary endpoints. Only patients who complete two surgical procedures with designated anesthesia regimens will be included in the final analyses.  Analyses will be performed using SPSS (version 19.0; IBM SPSS, Chicago, IL) and R software (version 3.6.0, R Foundation for Statistical Computing, Vienna, Austria) according to the prespecified analysis plan. |
| **Study Period** | From anesthesia clinic visit until hospital discharge |

# **Flow Chart**

|  | Screening | Study Phase | | | |
| --- | --- | --- | --- | --- | --- |
|  |  | Treatment | Post treatment follow-up | | |
|  | Anesthesia clinic visit | In OR | During anesthesia | Emergence from anesthesia | Hospital discharge |
| Evaluation | | | | | |
| Inclusion/Exclusion Criteria | 🞪 |  |  |  |  |
| Medical/Surgical History | 🞪 |  |  |  |  |
| Informed Consent | 🞪 |  |  |  |  |
| Prior Medication History | 🞪 |  |  |  |  |
| Patient Demographics | 🞪 |  |  |  |  |
| Clinical Examination | 🞪 |  |  |  |  |
| Vital Signs | 🞪 |  |  |  |  |
| Lab Testing | 🞪 |  |  |  |  |
| Randomization |  | 🞪 |  |  |  |
| Treatment | | | | | |
| Intervention |  | 🞪 |  |  |  |
| Compliance |  | 🞪 |  |  |  |
| Outcome evaluation | | | | | |
| Primary |  |  | 🞪 |  |  |
| Secondary |  |  | 🞪 | 🞪 | 🞪 |
| Safety | | | | | |
| AE/SAE (if any) |  |  | 🞪 | 🞪 | 🞪 |

AE, adverse event; SAE, serious adverse event.

# **Glossary**

- **Good Clinical Practice (GCP)**

An international standard of ethics and scientific quality to design, conduct, register and confront studies on human subjects. Adherence to these standards not only guarantees the safety, the well-being, and the rights of the participates, in accordance with the Helsinki Declaration (1947) principles, but also the reliability of the study data.

- **Ethic Committee (EC)**

An independent organization, composed by health care and non-heath care staff that is responsible for the safeguard of the safety, the well-being, and the rights of study subjects and that must also publicly guarantee this safeguard by, for example, expressing an opinion on the experimental protocol, on the investigators, on the adequacy of the structures, methods and documents used to inform the subjects and obtain informed consent.

- **Clinical Research Office (CRO)**

The Clinical Research Office takes care of the preparation and revision of all documents involved in research protocols (whether sponsored, internal, or part of a study group), including amendments and modifications, that are then analyzed by the EC at First Affiliated Hospital of Soochow University to be approved and then authorized. The CRO works as a scientific and technical secretariat of the EC.

- **No Profit Study**

No profit study does not aim at the industrial development of a drug or, in any case, not for profit, but aims at improving clinical practice. This objective must be guaranteed by the protocol relevance, by the peculiarity of the disease, and by the type of treatment.

- **Investigator**

A physician qualified in clinical research is responsible for this clinical trial in a university hospital. If the trial is followed by a group of investigators in the same hospital, the investigator who is responsible is called “Principal Investigator” (PI).

- **Case Report Form (CRF)**

The Case Report Form (CRF) is a printed or digital document designed to register all the information required by the study protocol that must be reported regarding each study subject.

- **Adverse Event (AE)**

Any negative clinical event that involves a patient undergoing a clinical trial who has received a study drug, even though the event does not necessarily have a causative correlation with the treatment.

- **Serious Adverse Event (SAE)**

Any adverse event or drug reaction that, regardless of the dose, causes the patient’s death or threatens the subject’s life, requires hospitalization, or prolongs hospitalization, or causes prolonged or severe invalidity, or involved a congenital anomaly or a birth problem.

# **Background and Rationale**

## Postoperative nausea and vomiting

Opioids are an important part of treatment of all types of pain, including moderate and severe pain, malignant and non-malignant pain, and chronic and acute pain [1,2]. However, due to the prevalence and abuse of opioids, the adverse reactions raised significant concerns, such as postoperative nausea and vomiting (PONV), respiratory depression, hyperalgesia, impaired gastrointestinal motility, et al. [3-7]. PONV is frequent in patients who undergo general anesthesia and surgery. PONV not only leads to significant distress of patients, but also is associated with an increased risk for postoperative morbidity and higher healthcare costs.

## Opioid-free anesthesia

In recent years, with the concept of enhanced recovery after surgery (ERAS) and "comfortable medical treatment", opioid free anesthesia (OFA) and analgesic techniques have been used. OFA is a multimodal anesthesia strategy using α-2 agonists, N-methyl-D-aspartate (NMDA) antagonists, and local anesthetics to replace opioids. OFA has been carried out in a variety of operations [8-9]. Bakan et al. [10] have shown the effectiveness of OFA in laparoscopic cholecystectomy. This anesthesia scheme can avoid pain, allergy, nausea and vomiting caused by opioids. Parsa et al. [11] showed that the use of OFA in breast plastic surgery significantly reduced PONV, shorten the discharge time and reduced the unplanned admission rate. However, there are still controversies on the application of OFA for various surgical procedures [12,13].

## The aim of this study

We design this randomized crossover trial to test the primary hypothesis that OFA leads to a lower incidence of PONV, when compared to a conventional opioid-inclusive anesthesia, in patients undergoing surgical treatments of lower extremity wounds. As for the secondary aims, we will explore the severity of PONV, postoperative pain outcomes, adverse effects, and recovery from anesthesia between the two groups.

# **Study Design**

## Description of this study

This is an investigator-initiated, single-center, prospective, randomized, double-blind, controlled crossover trial. Based on the crossover design, a same patient will receive one of the two anesthesia regimens (OFA and opioid-inclusive anesthesia) in a random sequence during separate surgical procedures. This study is conducted at a teaching hospital (The First Affiliated Hospital of Soochow University, Suzhou, Jiangsu, China).

After screening of eligibility, patients who meet the enrollment criteria will be randomized (1:1) to 1 of 2 treatment sequences: OFA followed by opioid-inclusive anesthesia (sequence 1) and opioid-inclusive followed by OFA (sequence 2). The randomization results will be stored in sealed opaque envelopes.

Consented patients will receive standard perioperative management and corresponding study interventions. The study medications (esketamine, lidocaine, and dexmedetomidine in the OFA regimen; sufentanil and normal saline in the opioid-inclusive anesthesia regimen) look identical. The anesthesia providers will be blinded to the group assignment. An independent trained investigator who is blinded to group allocation will assess the study outcome measures. The allocation and treatment codes will not be disclosed to patients or research personnel until the completion of final analysis.

## Duration of this study

Study team physicians are responsible for screening all patients undergoing a scheduled procedure. The number of eligible, consented, enrolled, and randomized patients is recorded in addition to the reasons for non-participation in the trial. During their hospital stay, patients are closely monitored and all outcomes are recorded. For this reason, missing endpoints are expected to be rare. The day before their appointment, the patient is contacted by a member of the study staff to remind them that a member of the team will collect research data during their visit. This is done by a phone call (or email if unreachable by phone). Based on the experience and our pilot data in our institution as well as the results of previous studies, it is estimated that it will take 6 months to enroll enough patients.

# **Study Outcomes**

## Primary outcome

- The incidence of PONV within the first 48 h postoperatively.

## Secondary outcomes

- Severity of PONV within 48 h postoperatively;
- Antiemetic rescue therapy;
- Postoperative pain scores within 48 h postoperatively, the worst pain, and need for rescue analgesia;
- Postoperative sedation;
- Hypotension, bradycardia, hypertension, tachycardia, and hypoxemia;
- Psychotomimetic or dissociative effects;
- Time to extubation;
- Length of post-anesthesia care unit stay.

# **Selection of Patients**

## Inclusion criteria

- Age ≥ 18 years old;
- American society of Anesthesiologists status I to III;
- Scheduled for two separate treatments for lower extremity wounds under general anesthesia;
- An interval between the procedures ≥ 5 days.

## Exclusion criteria

- Unplanned or emergency surgery;
- Severely infected or burn wounds;
- Left ventricular ejection fraction < 40%, second-degree or greater atrioventricular block, sick sinus syndrome, or severe bradycardia (heart rate < 50 beats/min);
- Severe liver or renal dysfunction (Child Pugh grade C or renal replacement therapy);
- Epilepsy or seizures;
- Preoperative use of sedatives or analgesics;
- Allergy to medications used in this study.

# **Study Treatment**

## 8.1 Randomization and blinding

An independent investigational pharmacist generates the random numbers using a computer-generated process, with an allocation ratio of 1:1 and permuted block sizes of 2 and 4. The details of randomization will be kept in sealed opaque envelopes. Patients will be randomized to 1 of 2 treatment sequences: OFA followed by opioid-inclusive anesthesia (sequence 1) and opioid-inclusive followed by OFA (sequence 2). The independent pharmacist formulates the study medications in the same fashion. These study medications (esketamine, lidocaine, and dexmedetomidine; sufentanil and normal saline) look identical. The anesthesia providers will be blinded to the group assignment. An independent trained investigator who is blinded to group allocation will assess the study outcome measures. The allocation and treatment codes will not be disclosed to patients or research personnel until the completion of final analysis.

In case of an emergency (e.g., unexpected rapid deterioration in the patient’s clinical status), attending anesthesiologists or endoscopists could request unmasking of the treatment allocation, or adjust drug administration if necessary. To maintain the overall quality, legitimacy, and integrity of the clinical trial, unblinding of the test drug may occur only in critical circumstances when severe adverse events happen and considered to be related to esketamine administration. In this circumstance, the PI. fully documents and explains the reasons for unblinding in a report to the Institutional Review Board (IRB).

## 8.2 Anesthesia and study interventions

Patients in sequence 1 will receive OFA during the first treatment procedure and opioid-inclusive anesthesia during the second procedure. Patients in sequence 2 will receive opioid-inclusive anesthesia during the first treatment procedure and OFA during the second procedure. The washout period between the two treatments is at least 5 days. Table 1 shows the detailed administration of study medications.

In the preoperative waiting area, the baseline HR and mean blood pressure (MBP) will be recorded. In the operating room, patients will be monitored with electrocardiography, noninvasive blood pressure, pulse oximetry (SpO2), and bispectral index (BIS). General anesthesia will be induced and maintained according to the anesthesia regimen. After anesthesia induction, i.v. cisatracurium 0.15 mg/kg will be given for tracheal intubation. The lungs will be ventilated with tidal volume 8–10 ml/kg, 12–18 breaths/min, inspired oxygen fraction 50–80% in air, and positive end-expiratory pressure 5–8 cmH2O to maintain SpO2 ≥95% and end-tidal carbon dioxide 35–45 mmHg. Propofol infusion will be titrated to maintain the BIS values within 40–60. Intraoperative analgesia will be provided with dexmedetomidine and esketamine in the OFA regimen or sufentanil in the opioid-inclusive anesthesia regimen. Cisatracurium will be administered for tracheal intubation only, without additional doses during surgery. Neuromuscular blockade reversal agents will not be routinely used. Local anesthesia will not be performed. Patients will be extubated in a post-anesthesia care unit (PACU). A modified Aldrete score ≥9 indicates readiness for PACU discharge to surgical wards.

Prophylaxis of PONV includes i.v. dexamethasone 5 mg after anesthesia induction and intraoperative ondansetron 4 mg. At PACU discharge and 24 and 48 h postoperatively, the severity of PONV will be rated as none, mild, moderate, or severe. To treat severe PONV, antiemetics with additional ondansetron 4 mg will be given. Patients will receive i.v. flurbiprofen axetil 50 mg at the end of surgery and every 12 h during the first two postoperative days, and oral acetaminophen will be given. Pain scores at 24 and 48 h postoperatively as well as the worst pain will be assessed using the numerical rating scale (NRS, 0–10; 0 = no pain, 10 = the most severe pain). If patients experience pain with NRS scores ≥ 4, rescue analgesia with i.v. nalbuphine will be administered.

**Table 1.** Details of study medications

|  | **Opioid-free anesthesia** | **Opioid-inclusive anesthesia** |
| --- | --- | --- |
| **Anesthesia induction** | Esketamine 0.2–0.4 mg/kg | Sufentanil 0.2–0.4 μg/kg |
|  | Lidocaine 1 mg/kg | Normal saline |
|  | Propofol 1.5–2.0 mg/kg | Propofol 1.5–2.0 mg/kg |
| **Anesthesia maintenance** | Dexmedetomidine 0.3–1.0 μg/kg/h | Normal saline infusion |
|  | Esketamine 0.1 mg/kg boluses | Sufentanil 0.1 μg/kg boluses |
|  | Propofol 2–10 mg/kg/h | Propofol 2–10 mg/kg/h |

## 8.3 Accountability and compliance

This study is primarily designed to see the effects of OFA on PONV in adult patients undergoing treatments for wounds of lower extremities. The principal investigator will be responsible that this trial is conducted as specified and in accordance with the applicable regulatory requirements. Jiangsu Hengrui Medicine Co, Ltd, Jiangsu, China helps to provide the blinded intervention drugs and placebos, but the pharmaceutical company will not participate in any other parts of the trial. All patients will receive standard perioperative treatment and the investigational drug will be delivered according to this prespecified standard protocol. The name and dosage of other relevant medications will be recorded in the Case Report Form.

# **Safety Concerns**

## Safety profile of OFA

Bakan and colleagues evaluated the use of OFA with dexmedetomidine, lidocaine, and propofol in patients undergoing laparoscopic cholecystectomy, showing that OFA reduced postoperative fentanyl consumption within two days after surgery, the incidence of PONV, and the need for rescue antiemetics. For patients undergoing bariatric surgery, the opioid-free total intravenous anesthesia with dexmedetomidine ketamine, and propofol reduced the prevalence and severity of PONV in the postoperative period. On the other hand, the possible drawbacks of OFA include increased risks of bradycardia, hypotension and sedation, a longer time to extubation, and a prolonged length of PACU stay. These adverse effects are often associated with a high dose of dexmedetomidine. In our study protocol, dexmedetomidine will be infused at a rate of 0.3–1.0 μg/kg/h without a loading dose, which may minimize the associated risks.

## Perioperative monitoring

In the preoperative waiting area, the baseline HR and mean blood pressure (MBP) will be recorded. In the operating room, patients will be monitored with electrocardiography, noninvasive blood pressure, pulse oximetry (SpO2), and bispectral index (BIS). Intraoperative propofol infusion will be titrated to maintain the BIS values within 40–60. After surgery, patients will be extubated in the PACU, and a modified Aldrete score ≥9 indicates readiness for PACU discharge to surgical wards.

During surgery and in the PACU, hypotension (a decrease in MBP > 30% of baseline value), bradycardia (HR < 50 beats/min), hypertension (an increase in MBP > 30% of baseline value), and tachycardia (HR > 100 beats/min) will be treated using ephedrine, phenylephrine, atropine, esmolol, or urapidil at the discretion of the attending anesthesiologist. Sedation will be assessed in the PACU using the Richmond Agitation Sedation Scale, with a score of ≤ -2 indicating postoperative sedation. Hypoxemia (SpO2 < 90% on room air) in the PACU and surgical ward will be managed with oxygen supplementation via a nasal catheter or mask ventilation if necessary.

## Discontinuation of intervention and unmasking

The criteria for patient discontinuation from the study are as follows:

1) Voluntary discontinuation by a patient;

2) Exiting the protocol for safety reasons based on the judgement of the clinical or research staff including acute worsening of vital signs.

In case of an emergency (e.g., unexpected rapid deterioration in the patient’s clinical status), attending anesthesiologists or endoscopist could request unmasking of the treatment allocation if necessary. To maintain the overall quality, legitimacy, and integrity of the clinical trial, unblinding of the test drug may occur only in critical circumstances when severe adverse events happen and considered to be related to esketamine administration. In this circumstance, the PI. fully documents and explains the reasons for unblinding in a report to the IRB.

# **Statistical Plan**

## Sample size

According to previous studies, that use of OFA, compared to opioid-inclusive anesthesia, reduced the incidence of PONV from 37.3% to 20% (an absolute reduction of 17.3%) following bariatric surgery [14] and from 33% to 13% (an absolute reduction of 20%) following laparoscopic cholecystectomy [10]. Our institutional data suggested that the incidence of PONV was approximately 30% in patients who had surgical wound treatment with opioid-inclusive anesthesia. We hypothesize that the OFA regimen would reduce the PONV incidence from 30% to 10%. We conducted the power analysis to show that 59 patients are needed in each group with α = 0.05 and power = 80%. Considering a possible dropout rate of 18%, we expand the sample size to 72 patients. The sample size calculation was performed using the PASS software (version 15.0.5, NCSS, LCC, Kaysville, UT).

## Statistical analysis

All data analyses will be carried out according to the pre-established analysis plan. Continuous variables will be tested for normal distribution using the Shapiro-Wilk test and presented as means ± standard deviations if normally distributed and medians (interquartile ranges) if not. The groups will be compared using the independent t-test and Mann–Whitney rank-sum test, as appropriate. Categorical variables will be presented as numbers (percentages) and analyzed using the Chi-squared test or Fisher exact test, as appropriate.

Demographic data and baseline characteristics will be presented using descriptive statistics only. For the primary and secondary endpoints, the treatment effects will be analyzed using odds ratio or mean difference with 95% confidence intervals. A mixed linear model or logistic regression model will be used to test for treatment differences with sequence, period, and treatment as fixed effects and subject as a random effect. In addition, we will perform analyses adjusting for two covariates (Apfel’s PONV risk scores and length of procedure). Subgroup analyses will be conducted for the primary outcome according to sex and PONV risk scores. We have no plan of multiple testing correction for the secondary endpoints, and thus these results should be interpreted as exploratory.

In this crossover trial, if a patient undergoes only one surgery after randomization, they will be regarded as dropouts. Patients who complete two surgical procedures with designated anesthesia regimens will be included in the final analyses. Statistical analyses will be performed using the SPSS (version 19.0; IBM SPSS, Chicago, IL) and R software (version 3.6.0, R Foundation for Statistical Computing, Vienna, Austria).

## Statisticians

Trial statistical analysis: Ya-juan Zhu, Ke Peng

Independent statistician: Yao-yu Ying (Department of Epidemiology and Biostatistics, School of Public Health, Medical College of Soochow University, Suzhou, Jiangsu, 215123, China)

# **Ethical Considerations**

## Ethical approval and clinical trial registration

The Ethics Committee of The First Affiliated Hospital of Soochow University will assess the protocol and the written and dated approval signed by the Ethics Committee chairman will be obtained. The study will be registered at the Chinese Clinical Trial Registry (www.chictr.org.cn). This study will be conducted in accordance with the principles laid down by the World Medical Assembly and all applicable amendments (Helsinki, 1964) and the ICH guidelines for Good Clinical Practice. This clinical trial will be conducted in compliance with international laws and regulations, and laws and regulations of China, as well as any applicable guidelines.

## Informed consent

The investigator (according to applicable regulatory requirements), or a person designated by the investigator, and under the investigator's responsibility, will fully inform the patient of all pertinent aspects of the clinical trial. All participants will be informed to the most fully extent about this study, in languages and terms they are able to understand. Prior to a patient’s participation in the clinical trial, he or she MUST sign the written Informed Consent Form. It will also be made clear to the patient that he or she can withdraw from the study at any time without giving reasons and that they will not be in any way disadvantaged by this. Any Informed Consent will be retained by the Investigator. A copy of the signed and dated written Informed Consent Form will be provided to the patient.

## Responsibilities

The investigators should perform the clinical trial in accordance with this clinical trial protocol, ICH/Good Clinical Practice, and the applicable regulatory requirements. The investigators ensure compliance with all procedures required by the clinical trial protocol and with all study required procedures. The investigator agrees to provide all information requested in the Case Report Forms (CRFs) in an accurate and legible manner.

# **Data Management and Clinical Trial Report**

## Source documents

According to the ICH/Good Clinical Practice, the monitoring team must check the Case Report Form (CRFs) entries and the source documents.

## Case Report Forms (CRFs)

It is the responsibility of the investigators to maintain adequate and accurate CRFs records. All CRFs will be completed electronically in their entirety to ensure accurate interpretation of data.

## Data protection

Data will be stored in the electronic database without indicating the name of the patients (a numeric code will be used).

## Clinical trial report

The principal investigator will be responsible for preparing a clinical trial report. When all data have been fully analyzed, the results of the clinical trial will be communicated to all investigators and to the Competent Authority.

# **References**

1. 17th WHO model list of essential medicines [http://www.who.int/medicines/areas/policy/world_medicines_situation/WMS_ch19_wAccess.pdf]. Accessed 7Oct 2016.

2. Rowbotham MC, Lindsey CD. How effective is long-term opioid therapy for chronic noncancer pain? Clin J Pain. 2007;23(4):300–302.

3. Harris JD. Management of expected and unexpected opioid-related side effects. Clin J Pain. 2008;24 Suppl 10: S8–13.

4. Cherny NI. Opioid analgesics: comparative features and prescribing guidelines. Drugs. 1996;51(5):713–737.

5. Chou R, Clark E, Helfand M. Comparative efficacy and safety of long-acting oral opioids for chronic non-cancer pain: a systematic review. J Pain Symptom Manage. 2003;26(5):1026–1048.

6. Moore RA, McQuay HJ. Prevalence of opioid adverse events in chronic non-malignant pain: systematic review of randomised trials of oral opioids. Arthritis Res Ther. 2005;7(5): R1046–1051.

7. Villars P, Dodd M, West C, Koetters T, Paul SM, Schumacher K, Tripathy D, Koo P, Miaskowski C. Differences in the prevalence and severity of side effects based on type of analgesic prescription in patients with chronic cancer pain. J Pain Symptom Manage. 2007;33(1):67–77.

8. Mulier J. Opioid free general anesthesia: a paradigm shift? Rev Esp Anestesiol Reanim,2017,64(8):427–430.

9. Sultana A, Torres D, Schumann R. Special indications for Opioid Free Anaesthesia and Analgesia, patient and procedure related: Including obesity, sleep apnoea, chronic obstructive pulmonary disease, complex regional pain syndromes, opioid addiction and cancer surgery. Best Pract Res Clin Anaesthesiol,2017,31(4): 547–560.

10. Bakan M, Umutoglu T, Topuz U, et al. Opioid-free total intravenous anesthesia with propofol, dexmedetomidine and lidocaine infusions for laparoscopic cholecystectomy: a prospective, randomized, double-blinded study. Braz J Anesthesiol. 2015;65(3):191–199.

11. Parsa FD, Cheng J, Stephan B, et al. Bilateral breast reduction without opioid analgesics: a comparative study. Aesthet Surg J,2017,37(8):892–899.

12. Massoth C, Schwellenbach J, Saadat-Gilani K, et al. Impact of opioid-free anaesthesia on postoperative nausea, vomiting and pain after gynaecological laparoscopy - A randomised controlled trial. J Clin Anesth. 2021;75:110437.

13. Beloeil H, Garot M, Lebuffe G, et al. Balanced Opioid-free Anesthesia with Dexmedetomidine versus Balanced Anesthesia with Remifentanil for Major or Intermediate Noncardiac Surgery. Anesthesiology. 2021;134(4):541–551.

14. Ziemann-Gimmel P, Goldfarb AA, Koppman J, Marema RT. Opioid-free total intravenous anaesthesia reduces postoperative nausea and vomiting in bariatric surgery beyond triple prophylaxis. British journal of anaesthesia. 2014;112(5):906–911.
